# Whole-genome sequencing to characterize the genetic structure and transmission risk of *Mycobacterium tuberculosis* in Yichang city of China

**DOI:** 10.3389/fpubh.2022.1047965

**Published:** 2023-01-09

**Authors:** Lv Ji, Feng-Xi Tao, Yun-Fang Yu, Jian-Hua Liu, Feng-Hua Yu, Chun-Lin Bai, Zheng-Yang Wan, Xiao-Bo Yang, Jing Ma, Pan Zhou, Zhao Niu, Ping Zhou, Hong Xiang, Ming Chen, Zhou Xiang, Fang-Qiong Zhang, Qi Jiang, Xiao-Jun Liu

**Affiliations:** ^1^Institute of Public Health Inspection, Yichang Center for Disease Control and Prevention, Yichang, Hubei, China; ^2^School of Public Health, Wuhan University, Wuhan, Hubei, China; ^3^Institute of Infectious Disease Prevention and Control, Yichang Center for Disease Control and Prevention, Yichang, Hubei, China; ^4^Institute of Infectious Disease Prevention and Control, Yidu Center for Disease Control and Prevention, Yidu, Hubei, China; ^5^Clinical Laboratory, Yidu First People's Hospital, Yidu, Hubei, China; ^6^Institute of Infectious Disease Prevention and Control, Zigui Center for Disease Control and Prevention, Zigui, Hubei, China; ^7^Clinical Laboratory, Zigui County People's Hospital, Zigui, Hubei, China

**Keywords:** *Mycobacterium tuberculosis*, multidrug-resistant tuberculosis, whole genome sequencing, transmission, Chinese rural area

## Abstract

**Objective:**

The burden of both general and drug-resistant tuberculosis in rural areas is higher than that in urban areas in China. To characterize the genetic structure and transmission risk of *Mycobacterium tuberculosis* in rural China, we used whole genome sequencing to analyze clinical strains collected from patients in two counties of Yichang for three consecutive years.

**Methods:**

From 2018 to 2020, sputum samples were collected for cultures from patients with suspected tuberculosis in Yidu and Zigui county, and DNA was extracted from the positive strains for genome sequencing. The online SAM-TB platform was used to identify the genotypes and drug resistance-related mutations of each strain, establish a phylogenetic tree, and calculated the genetic distances between pairwise strains. Twelve single nucleotide polymorphisms (SNPs) were used as thresholds to identify transmission clusters. The risk of related factors was estimated by univariable and multivariable logistic regression.

**Results:**

A total of 161 out of the collected 231 positive strains were enrolled for analysis, excluding non-tuberculous mycobacterium and duplicate strains from the same patient. These strains belonged to Lineage 2 (92, 57.1%) and Lineage 4 (69, 42.9%), respectively. A total of 49 (30.4%) strains were detected with known drug resistance-related mutations, including 6 (3.7%) multidrug-resistant-TB (MDR-TB) strains and 11 (6.8%) RIF-resistant INH-susceptible TB (Rr-TB) strains. Six of the MDR/Rr-TB (35.3%) were also resistant to fluoroquinolones, which made them pre-extensively drug-resistant TB (pre-XDR-TB). There were another seven strains with mono-resistance to fluoroquinolones and one strain with resistance to both INH and fluoroquinolones, making the overall rate of fluoroquinolones resistance 8.7% (14/161). A total of 50 strains (31.1%) were identified as transmission clusters. Patients under 45 years old (adjusted odds ratio 3.46 [95% confidential intervals 1.28–9.35]), treatment-naive patients (6.14 [1.39–27.07]) and patients infected by lineage 4 strains (2.22 [1.00–4.91]) had a higher risk of transmission.

**Conclusion:**

The drug resistance of tuberculosis in rural China, especially to the second-line drug fluoroquinolones, is relatively serious. The standardized treatment for patients and the clinical use of fluoroquinolones warrant attention. At the same time, the recent transmission risk of tuberculosis is high, and rapid diagnosis and treatment management at the primary care needs to be strengthened.

## Introduction

Tuberculosis (TB) caused by *Mycobacterium tuberculosis* (MTB) infection is currently the second largest single source of infection after Corona Virus Disease 2019 (COVID-19) (1). Due to the irregular use of anti-TB drugs and the characteristics of the cell wall structure of MTB, MTB has produced varying degrees of resistance to anti-TB drugs (2). Multidrug-resistant TB (MDR-TB) is resistant to the two both major first-line drugs, rifampicin (RIF) and isoniazid (INH), requires a treatment cycle of up to 2 years, but the cure rate is only 59% (3). At the same time, China is a high-burden country of TB and MDR-TB, with 800,000 new cases every year, approximately 50,000 of which are MDR-TB cases (3). According to statistics, about 71% of TB patients in China live in rural areas (4, 5), and the prevalence of drug-resistant TB in rural areas is significantly higher than the national level (6). In the investigation of second-line anti-TB drugs in rural areas, it was found that strains resistant to INH and fluoroquinolones (FQs) were easier to spread than drug-sensitive strains (7). Financial constraints and weak public health infrastructure lead to the delay of patients' clinic visits, which leads to delays in diagnosis. In addition, the use of inappropriate anti-TB drugs, the easy availability of second-line anti-TB drugs and the relatively poor follow-up of patients during treatment all lead to the high incidence of drug-resistant TB in rural areas.

Infection with MTB develops into TB primarily through two mechanisms: recent transmission events and potential *in vivo* reactivation of TB (8, 9). Molecular epidemiological studies have shown that TB caused by the spread of TB strains may account for 35 to 40% (even as high as 70%) of TB cases (10). Increasing evidence shows that the spread of drug-resistant TB strains is an important driving factor for the epidemic of TB (11, 12). In view of the transmission of MTB, previous studies have mostly focused on economically developed areas such as Shanghai and Shenzhen, where 73 and 25.2% of MDR patients were likely to be transmitted, respectively (13, 14). However, as a high incidence of TB, less relevant research on the transmission of MDR-TB is available for rural areas with underdeveloped economies and a lack of health resources.

To study the characteristics of prevalent TB in rural areas and analyze the risk factors for transmission, we conducted a prospective molecular epidemiological study in two counties of Yichang city, China. The registered incidence of active pulmonary TB in Yichang was 84.7/100,000, and a previous cross-sectional investigation from 2017 to 2018 showed a high resistance rate among pulmonary TB in Yichang city, with the proportions of bacteria-positive TB with resistance to INH and RIF being 16.23 and 7.55%, respectively (15, 16). This study employed whole-genome sequencing (WGS) technology to analyze the full drug-resistance profiles of all the anti-TB drugs and characterize the genetic structure of prevalent strains. Transmission clusters were identified based on pairwise comparisons of bacteria genome sequences and analyzed for the risk factors.

## Methods

### Study setting and sample inclusion

Yichang is the third largest prefecture-level city of Hubei province, China, with the land area of 21 thousand square kilometers and residential population of four million. From the administrated five districts and eight counties, we selected two representative counties Yidu and Zigui to study the molecular epidemiology of prevalent TB in rural areas. The study areas covered a population of 692,000, consisting 17.22 and 29.68% of the total population the rural population in the city, respectively.

From 2018 to 2020, a total of 1981 patients were diagnosed with active pulmonary TB in Yichang, and 42.3% (838/1981) of them were confirmed by bacteriological examinations. Generally, smear-positive patients and patients who were highly suspected of drug resistance would be tested by drug-susceptibility test. Overall, 45 patients were diagnosed and registered as MDR-TB, consisting 5.37% of the bacteriologic-positive patients. The counties had a relatively lower rate of detected MDR-TB, with the proportions of bacteria-positive TB being 2.85% (7/246) and 1.47% (6/408) in Yidu and Zigui, respectively. Clinical strains from positive cultures were sent for whole-genome sequencing to detect full drug-resistance profiles. We in total collected 231 strains from 161 patients, which consisted of 24.62% (161/654) of the bacteria-positive patients in the two counties during study period.

The basic demographic and clinical information of the enrolled patients was derived from the National Registration System of TB, including the patients' gender, age, nationality, occupation, living address and the treatment history of TB, et al.

### Whole genome sequencing and analysis

The genomic DNA of the culture-positive strains was extracted by the a cetyl trimethyl ammonium bromide (CTAB) method, and a 300 bp double-end sequencing library was established. The whole genome was sequenced on the Illumina 2,500 platform and analyzed using the online platform SAM-TB (17) (http://samtb.szmbzx.com) to identify the genotypes of TB strain and predict the drug resistance spectrum of each strain to 14 types of anti-TB drugs. The phylogenetic tree was established by the maximum likelihood method, and visualized with iTOL (https://itol.embl.de/). The raw data of whole-genome sequences were deposited in the China National Center for Bio-information (https://ngdc.cncb.ac.cn/) under the project number PRJCA013147.

### Epidemiological investigation of transmission clusters

The pairwise genetic distance between any two strains was calculated. The isolates with genetic distances less than 12 single nucleotide polymorphisms (SNPs) were considered as transmission clusters. Patients of the transmission clusters were further investigated epidemiologically by community physicians. The patients' residential address, workplaces, and frequently visited places were collected to identify the possible transmission sites of the clusters. Patients in each cluster were classified as transmission with confirmed epidemiological links if they had any shared visiting place or close contact. All the raw questionnaires were anonymized and associated with the patient's basic information by the TB registration number for subsequent statistical analysis. The investigation was conducted after the patients' oral consent, and the procedure was reviewed and consent by the Ethics Committee of Yichang Center for Disease Control and Prevention (No. LLSC201801).

### Related definitions

Strains resistant to both INH and RIF drugs were defined as MDR-TB, while strains that were resistant to only one of the two drugs were defined as Hr-TB and Rr-TB, respectively. According to the new definitions posed by WHO in 2020, MDR/Rr-TB plus resistance to FQs is defined as pre-extensively drug-resistant TB (pre-XDR-TB). Additional resistance to bedaquiline (BDQ) or linezolid (LZD) is defined as extensively drug-resistant TB (XDR-TB). Diagnostic delay was defined as the time from the self-reported onset of related symptoms by the patients and the diagnosis of TB in the designated hospitals.

### Statistical analysis methods

Excel was used to store and clean the data, and R (version 4.0.3) was used for all the statistical analysis. The differences of categorized variables between groups were compared by the *chi*-square test, and the risk of relevant factors for the transmission clusters were identified and the odds ratio (OR) and 95% confidential intervals (95% CI) of each risk factor were calculated by univariable and multivariable logistic regression. The difference was considered statistically significant at a criterion of *P* < 0.05.

## Results

### Basic characteristics of studied patients

**Figure 1:**
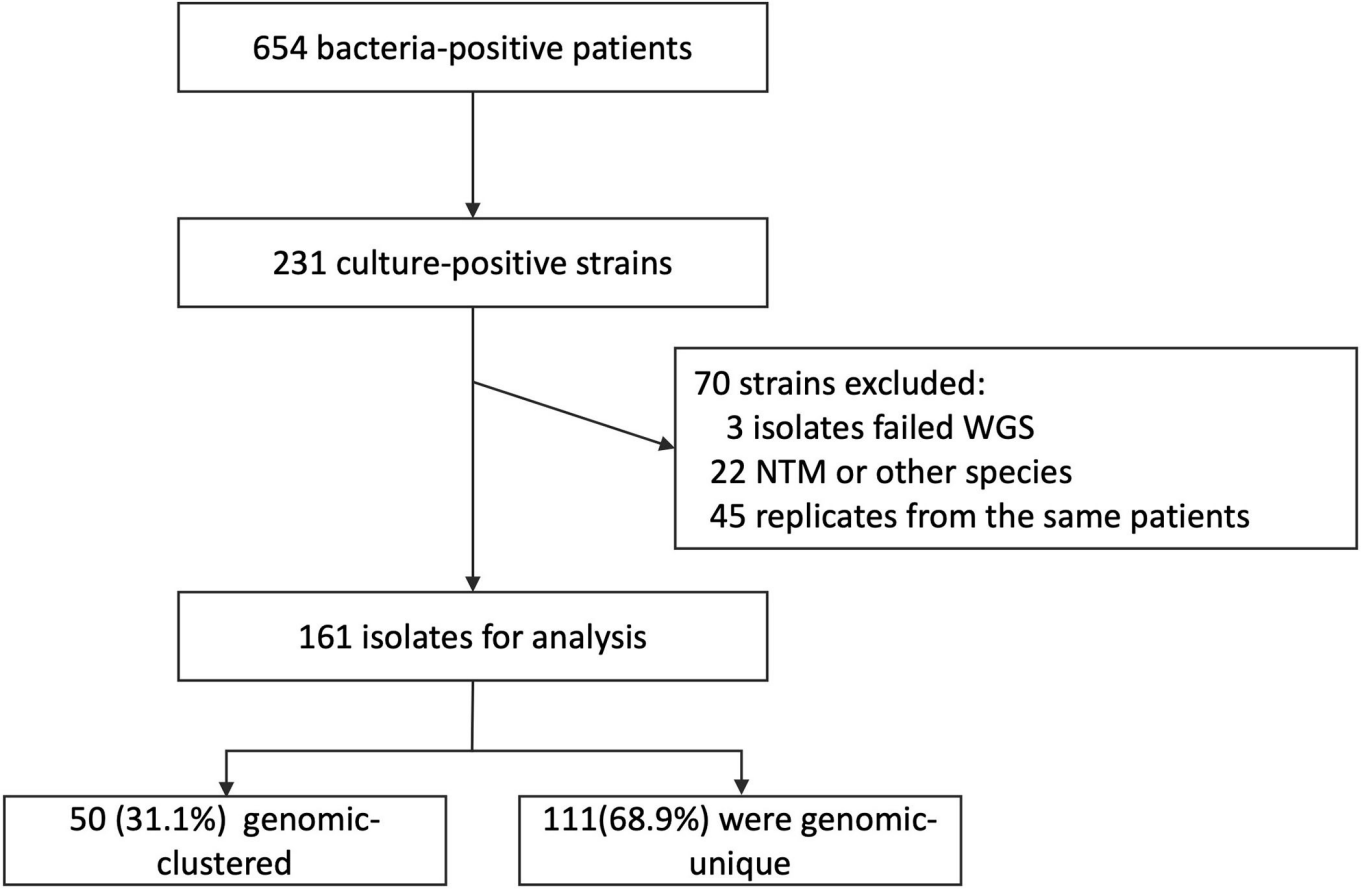
Patient inclusion flowchart.

From July 1, 2018 to December 31, 2020, a total of 654 patients were diagnosed of bacteria-positive pulmonary TB in Yidu and Zigui counties, with the reported incidence of 37.8 confirmed cases per 100,000 population per year. From the patients, 231 sputum samples were culture-positive and sent for WGS. Excluding 47 strains that were duplicate strains from the same patient, 184 representative strains were obtained. Among them, 18 strains were identified as non-tuberculous mycobacteria, 4 strains were of other species, and 1 strain failed to be sequenced. Finally, clinical strains from 161 TB patients and their clinical data were included in further analysis of molecular characteristics (Figure 1). The demographic characteristics of all the patients and sequenced patients were presented in Table 1. Totally, 24.62% (161/654) patients had positive cultures and were successfully sequenced of their clinical strains. Smear-negative patients were more likely to be performed of culture testing and enrolled in further analysis than smear-positive patients.

**Table 1:** Basic characteristics of tuberculosis patients during 2018–2020 in Yidu and Zigui, Yichang.

	**All bacteria-positive patients** ***N =* 654**	**Patients with whole-genome sequences** ***N =* 161^†^**	**Enrolled Yidu Patients** ***N =* 83**	**Enrolled** **Zigui Patients** ***N =* 75**
**Sex**				
Male	454 (69.4)	120 (74.5)	64 (77.1)	54 (72.0)
Female	200 (30.6)	41 (25.5)	19 (22.9)	21 (28.0)
**Age, years**				
≤ 45	112 (17.2)	27 (16.8)	14 (16.9)	12 (16.0)
46–60	208 (31.8)	45 (28.0)	22 (26.5)	21 (28.0)
61–75	250 (38.2)	64 (39.8)	33 (39.8)	31 (41.3)
>75	84 (12.8)	25 (15.5)	14 (16.9)	11 (14.7)
**Occupation** ^*§^				
Farmer	509 (77.8)	110 (68.3)	44 (53.0)	66 (88.0)
Other	145 (22.2)	51 (31.7)	39 (47.0)	9 (12.0)
**Treatment history**				
Treatment-naive	566 (86.5)	139 (86.3)	68 (81.9)	69 (92.0)
Retreated	88 (13.5)	22 (13.7)	15 (18.1)	6 (8.0)
**Diagnosis delay** ^**§**^				
≤ 2 weeks	215 (32.9)	50 (31.1)	18 (21.7)	32 (42.7)
2–4 weeks	197 (30.1)	41 (25.5)	10 (12.0)	29 (38.7)
4–8 weeks	91 (13.9)	30 (18.6)	24 (28.9)	5 (6.7)
>8 weeks	151 (23.1)	36 (22.4)	30 (36.1)	6 (8.0)
**Sputum smear** ^*^				
Negative	98 (15.0)	36 (22.4)	19 (22.9)	15 (20.0)
Positive	556 (85.0)	125 (77.6)	64 (77.1)	60 (80.0)
**Treatment outcome** ^*^				
Favorable	464 (70.6)	112 (69.6)	63 (75.9)	46 (61.3)
Unfavorable	97 (14.8)	42 (26.1)	18 (21.7)	24 (32.0)
Under treatment	95 (14.6)	7 (4.3)	2 (2.4)	5 (6.7)
**Strain genotype**				
Beijing	-	92 (57.1)	49 (59.0)	42 (56.0)
Euro-American	-	69 (42.9)	34 (41.0)	33 (44.0)
**Genomic clustering** ^**§**^				
Yes	-	50 (31.1)	37 (44.6)	11 (14.7)
No	-	111 (68.9)	46 (55.4)	64 (85.3)
**Rifampicin genetic resistance**				
Rifampicin sensitive	-	144 (89.4)	73 (88.0)	68 (90.7)
MDR/Rr-TB	-	17 (10.6)	10 (12.0)	7 (9.3)

The distributions of the variables were statistically different between all patients and sequenced patients (^*^), or between enrolled samples from Yidu and Zigui (§), with *P* value of *chi*-square test < 0.05;

† Three patients lived other districts than the two study sites.

Among the patients with whole-genome sequences of clinical strains, 75 cases (43.2%) were from Zigui, 83 cases (54.1%) were from Yidu, and the remaining 3 cases lived in other areas of the city. Male patients accounted for 74.5% (120/161) and the median age was 62 years old (interquartile range: 50–71); most of the patients were farmers, accounting for 68.3% (110/161). The number of farmers in Zigui was significantly higher than that in the Yidu (88 vs. 53%). The median diagnostic delay time of patients was 23 days (interquartile range: 12.0–47.5). Most patients (77.6%, 125/161) were smear-positive and 13.7% (22/161) were retreated patients.

### Genetic structure and drug resistance

**Figure 2:**
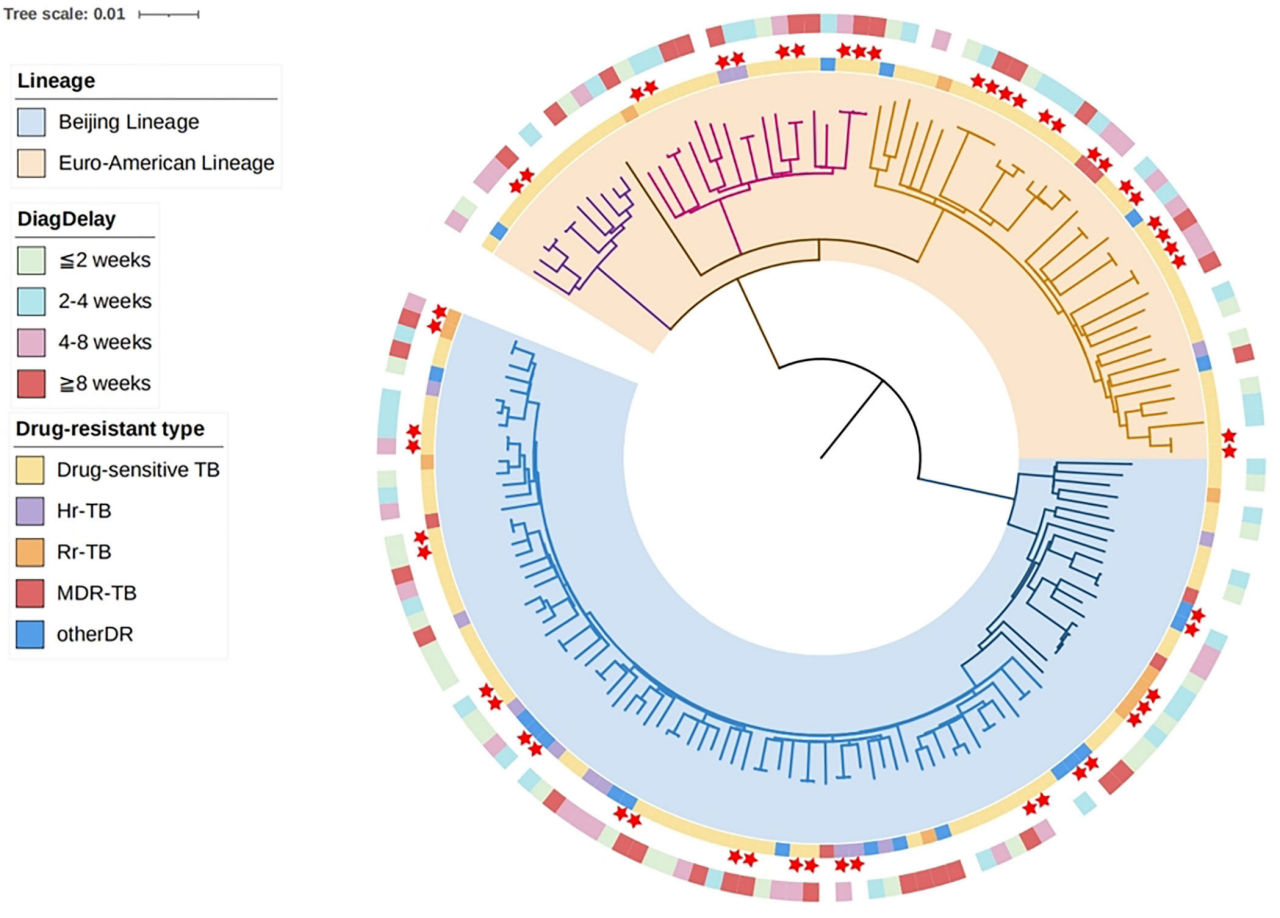
Phylogenetic tree and drug resistance spectrum of 161 tuberculosis strains in Yichang.

A phylogenetic tree was constructed based on the whole-genome sequences of the 161 *M*. tuberculosis strains of Yichang (Figure 2). The majority (57.1%, 92/161) belonged to Lineage 2, i.e., Beijing genotype strains, three quarters of which (77.2%, 71/92) belonged to the modern-Beijing sub-lineage (L2.3). The remaining 69 strains (42.9%, 69/161) were all Lineage 4 strains, also known as the Euro- American lineage, including 11 strains of L4.2, 2 of L4.3, 19 of L4.4, and 37 of L4.5 sub-lineages.

SAM-TB was used to identify the known gene mutations related to the drug resistance to 14 anti-TB drugs for each strain and predict its drug resistance spectrum, as shown in Figure 2. A total of 49 strains were detected with drug resistance-related gene mutations, and the arbitrary drug resistance rate was 30.4% (49/161). Six of which (3.7%, 6/161) were MDR-TB, and 11 (6.8%, 11/161) were resistant to RIF and susceptible to INH (Rr-TB), making the rate of MDR/Rr-TB 10.6% (17/161). The remaining drug-resistant strains included 13 (8.1%) INH-resistant RIF-susceptible (Hr-TB) strains, and strains with mono-resistance to FQs (7, 4.3%), SM (10, 6.2%), or PAS (2, 1.2%). It is worth noting that the resistance to second-line FQs were unexpectedly high, with the resistance rates among MDR/Rr-TB and RIF-susceptible strains of respectively 35.3% (6/17) and 5.6% (8/144).

In general, the drug resistance rates of different drugs from high to low were as follows: streptomycin (SM, 23 [14.3%]), INH (19 [11.8%]), RIF (17 [10.6%]), FQs (14 [8.7%]), ethionamide (ETO, 7 [4.3%]), para-aminosalicylic acid (PAS, 6 [3.7%]), pyrazinamide (PZA, 6 [3.7%]), ethambutol (EMB, 4 [2.5%]), aminoglycosides (AGs, 2 [1.2%]), and cycloserine (CS, 1 [0.6%]). No resistant strains to other second-line class A drugs were found. The resistance rate of Beijing lineage strains was significantly higher than that of Euro-American lineage strains, with arbitrary resistance rates of 40.2% (37/92) and 17.4% (12/69), respectively (Supplementary Table 1).

### Distribution of drug resistance spectrum and related factors

Among the 161 patients, retreated patients accounted for 13.7% (22/161). Most retreated patients (54.5%) had a diagnosis delay of < 2 weeks, while most treatment-naive patients (57%) had a diagnosis delay of < 4 weeks. Compared with treatment-naive patients, only 45.5% (45.5 vs. 77.3%) of retreated patients had a favorable treatment outcome (Supplementary Table 2). The drug resistance rate of retreated patients was significantly higher than that of new cases, and the arbitrary drug resistance rates were 40.9% (9/22) and 28.8% (40/139), respectively. Among the MDR-TB patients, the proportion of retreated patients was as high as 66.7% (4/6). It is worth noting that in addition to first-line drugs, retreated patients were also more likely to develop drug resistance to FQs and AGs (Table 2).

**Table 2:** Drug resistance spectrum of primary and retreated tuberculosis patients.

	**Total** ***N =* 161 (%)**	**Treatment-naive** ***N =* 139 (%)**	**Previously treated** ***N =* 22 (%)**	**χ^2^**	***P* value**
**Drug-resistance type**				17.01	0.002
DS	112 (69.6)	99 (71.2)	13 (59.1)	-	-
DR	19 (11.8)	16 (11.5)	3 (13.6)	-	-
Hr	13 (8.1)	13 (9.4)	0 (0)	-	-
Rr	11 (6.8)	9 (6.5)	2 (9.1)	-	-
MDR	6 (3.7)	2 (1.4)	4 (18.2)	-	-
**First-line drugs**					
INH	19 (11.8)	15 (10.8)	4 (18.2)	0.997	0.318
RIF	17 (10.6)	11 (7.9)	6 (27.3)	7.537	0.006
EMB	4 (2.5)	1 (0.7)	3 (13.6)	-	0.008
PZA	6 (3.7)	2 (1.4)	4 (18.2)	-	0.003
SM	23 (14.3)	18 (12.9)	5 (22.7)	1.483	0.223
**Second-line drugs** ^*^					
FQs	14 (8.7)	7 (5.0)	7 (31.8)	17.16	< 0.001
AGs	2 (1.2)	0 (0)	2 (9.1)	-	0.018
PAS	6 (3.7)	4 (2.9)	2 (9.1)	-	0.190
ETO	7 (4.3)	5 (3.6)	2 (9.1)	-	0.245
CS	1 (0.6)	0 (0)	1 (4.5)	-	0.137

* No resistance-conferring mutations were identified for linezolid, bedaquiline, clofazimine, or delamanid. *P* value was calculated by the *chi*-square test or the Fisher's exact test if the minimum theoretical frequency in chi-square test was < 1. DS, Drug-Susceptible; DR, Drug Resistance to other drugs beyond isoniazid and rifampicin; Hr, isoniazid-resistant rifampicin-susceptible; Rr, rifampicin-resistant isoniazid-susceptible; MDR, MultiDrug-resistant; INH, isoniazid; RIF, rifampicin; EMB, ethambutol; PZA, pyrazinamide; SM, streptomycin; FQs, fluoroquinolones; AGs, injectable aminoglycosides including amikacin, kanamycin, and capreomycin; PAS, para-aminosalicylic acid; ETO, ethionamide; CS, cycloserine.

### Genetic distance and clustering analysis

To analyze the transmission characteristics of TB in the study areas, the whole genome sequences of pairwise two strains were compared, and the genetic distance was calculated. When using 12-SNP as the threshold to identify transmission clusters, a total of 50 strains were divided into 23 transmission clusters, and the gene composition cluster rate was 31.1% (50/161).

To analyze the characteristics of TB strains that were more prone to transmission, we compared the demographic and pathogenic characteristics of clustered and non-clustered strains (Table 3). The results showed that the strains in patients under 45 years old were easier to clustering than that of other age groups. The clustering rate in the Yidu was significantly higher than that in the Zigui (44.6 vs. 14.7%). Patients whose diagnostic delay time was 4–8 weeks had the highest probability of genetic clustering (50%). There was no significant difference between the clustering rates among TB strains of different genotypes or drug-resistance profiles (Supplementary Table 3), except that the resistance rate of RIF in clustered strains was slightly higher than that of non-clustered strains (14.0 vs. 9.0%).

**Table 3:** Demographic and pathogenic characteristics of genomic-clustered and non-clustered tuberculosis.

	**Total** ***N =* 161**	**Genomic-** **clustered** ***N =* 50**	**Genomic-** **unique** ***N =* 111**	**χ^2^**	***P* value**
**Sex**				1.141	0.285
Female	41 (25.5)	10 (20.0)	31 (27.9)		
Male	120 (74.5)	40 (80.0)	80 (72.1)		
**Age, years**				12.215	0.007
≤ 45	27 (16.8)	15 (30.0)	12 (10.8)		
46–60	45 (28.0)	12 (24.0)	33 (29.7)		
61–75	64 (39.8)	20 (40.0)	44 (39.6)		
>75	25 (15.5)	3 (6.0)	22 (19.8)		
**Occupation**				1.928	0.395
Farmer	110 (68.3)	31 (62.0)	79 (71.2)		
Other	11 (6.8)	5 (10.0)	6 (5.4)		
Unemployment	40 (24.8)	14 (28.0)	26 (23.4)		
**Region**				16.666	< 0.001
Zigui	75 (46.6)	11(22.0)	64 (57.7)		
Yidu	83 (51.6)	37 (74.0)	46 (41.4)		
**Treatment history**				3.611	0.057
Treatment-naive	139 (86.3)	47 (94.0)	92 (82.9)		
Previously treated	22 (13.7)	3 (6.0)	19 (17.1)		
**Delayed diagnosis**				9.920	0.019
≤ 2 weeks	50 (31.8)	9 (18.0)	41 (38.3)		
2–4 weeks	41 (26.1)	12 (24.0)	29 (27.1)		
4–8 weeks	30 (19.1)	15 (30.0)	15 (14.0)		
>8 weeks	36 (22.9)	14 (28.0)	22 (20.6)		
**Sputum smear results**				0.553	0.457
Negative	36 (22.4)	13 (26.0)	23 (20.7)		
Positive	125 (77.6)	37 (74.0)	88 (79.3)		
**Treatment outcome**				4.341	0.037
Favorable	112 (72.7)	41 (83.7)	71(67.6)		
Other	42 (27.3)	8 (16.3)	34 (32.4)		

Logistic regression was used to calculate the adjusted odds ratio (aOR) that each factor increased the risk of recent transmission of TB, as shown in Table 4. In the univariate logistic regression model adjusted by the strain genotype and RIF resistance, patients under 45 years old were more likely to be infected with clustered strains, and their transmission risk was 3.46 (1.45–8.26) times higher than that of elder patients. The risk of treatment-naive patients was 4.09 (1.08–15.45) times higher than that in retreated patients. When the diagnostic delay was >4 weeks, the risk of transmission increased by 2.63 (1.31–5.30) times. The clustering rate of clinical strains in the Yidu area was significantly higher than that in Zigui, which were 44.6% (37/83) and 14.7% (11/75), respectively. The clustering rate of Euro-American strains was higher than that of Beijing strains (39.1 vs. 25.0%). The above related factors were included in the multivariable logistic regression model. The results showed that the risk factors independently related to the recent transmission of TB included patients aged ≤ 45 years (aOR 3.46; 95% CI 1.28–9.35), newly treated patients (aOR 6.14; 95% CI 1.39–27.07), Yidu region (aOR 6.45; 95% CI 2.37–17.54) and Euro-American strains (aOR 2.22; 95% CI 1.00–4.91), while RIF-resistant strains had no significant transmission advantage.

**Table 4:** Hierarchical multivariable logistic regression of risk factors for tuberculous transmission clusters.

**Risk factors**		Univariate regression^*^	**Multivariate regression**	**Multivariate regression of Zigui**	**Multivariate regression of Yidu**
Age ≤ 45 yeas old	aOR (95%CI)	3.46 (1.45–8.26)	3.46 (1.28–9.35)	4.63 (0.94–22.69)	4.02 (1.01–16.06)
	*p*-value	0.005	0.014	0.059	0.049
New cases	aOR (95%CI)	4.09 (1.08–15.45)	6.14 (1.39–27.07)	-	5.69 (1.20–26.96)
	*p*-value	0.038	0.016	-	0.028
Delayed diagnosis > 4 weeks	aOR (95%CI)	2.63 (1.31–5.30)	1.02 (0.41,2.50)	-	-
	*p*-value	0.007	0.973	-	-
Samples in Yidu	aOR (95%CI)	4.92 (2.24–10.82)	6.45 (2.37–17.54)	-	-
	*p*-value	< 0.001	< 0.001	-	-
Euro-American lineage	aOR (95%CI)	2.063 (1.04–4.11)	2.22 (1.00–4.91)	1.20 (0.29–5.03)	2.95 (1.10–7.90)
	*p*-value	0.04	0.049	0.804	0.032
MDR/RR-TB	aOR (95%CI)	1.96 (0.68–5.66)	2.57 (0.70,9.42)	4.95 (0.52–47.39)	2.10 (0.38–11.69)
	*p*-value	0.212	0.156	0.166	0.397

* Univariate regression corrected genotype and RFP resistance, and calculated adjusted odds ratio (aOR) of each patient's clinical variables and 95% confidential intervals (95% CI).

### Spatial and social associations of clustered patients

**Figure 3:**
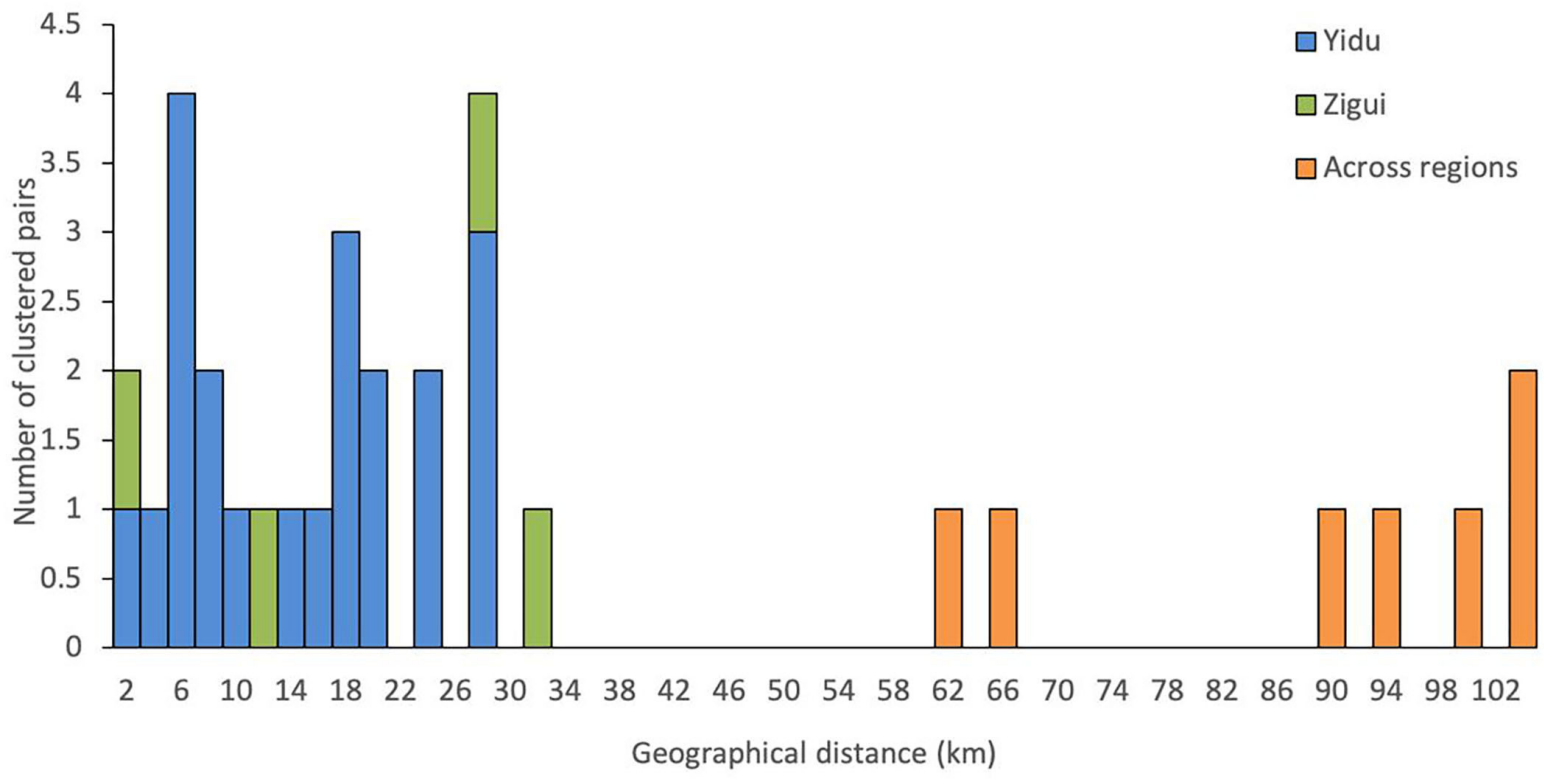
Spatial and social association of tuberculosis patients with transmission clusters.

The resident addresses of clustered patients were connected, and the living distance was calculated, with the distribution shown in Figure 3. Most of the clustered cases in the same county lived within 32 kilometers. Six pairs of clustered cases were found to live in different counties with a distance of over 60 kilometers. If the 2-km range is regarded as the same community, only 2 pairs (8%, 4/50) of the 50 clustered patients lived in the same community.

The epidemiological investigation of cluster cases identified confirmed epidemiological links in only two clusters. Two patients in one cluster did not know each other but lived on the same street with a geographic distance within 2 km. The former patient developed clinical symptoms in October 2019 and was diagnosed with TB 1 month later. The latter developed symptoms and diagnosed with TB 1 year later. The two patients in another cluster knew each other, lived in the same village and often participated in weddings and funerals in the village together. There was an approximate 3-month interval between their onsets of clinical symptoms, while the diagnosis of TB was in the same month.

## Discussion

To our knowledge, this is the first genomic epidemiological study in the rural areas of Hubei Province, China. Based on the whole-genome sequencing analysis of 161 patients, we characterized the genetic structure of prevalent TB strains with their full profiles of drug resistance, and offered a glance at the TB transmission pattern with risk factors in rural China. The results showed high levels of drug resistance among bacteria-positive TB, especially, for resistance to INH (11.8%), RIF (10.6%), and FQs (8.7%). Though the samples consisted of only one fourth of total bacteria-positive cases, 31.1% of the strains were genomic-clustered and formed into 23 transmission clusters. Patients less than 45 years old, new cases, and patients had a diagnosis delay of more than 4 weeks presented higher risk of TB transmission.

We detected over 10% of the bacteria-positive TB to be resistant to RIF which made the cases MDR/RR-TB. The rate was relatively higher than the national average of 7.4% (18), but similar to the level of several areas in rural China with an MDR-TB rate of 11.3% (7). Specifically, the drug resistance rate of FQs reached 8.7% among general TB, and even as many as 83.3% among MDR-TB in rural Yichang. The rates were much higher than previous reports of the national investigation in China with approximately 30% of MDR-TB gaining further resistance to FQs (19), and central and western China with high TB burdens such as Hunan (20) and Chongqing (21), where about 34.0 and 41.0% of MDR-TB developed into pre-XDR-TB, respectively. In rural areas, high levels of FQ resistance, especially, unexpected monoresistance, indicated the irregular clinical usage of the broad-spectrum antibiotics including FQ drugs. These drugs are widely used to treat bacterial infections in the respiratory, gastrointestinal and urinary tracts (22), and they can be purchased in most pharmacies in China without a prescription. The accessibility increased overuses of antibiotics and could be the possible reasons for monoresistance of TB.

Moreover, compared with cities, the rate of inappropriate prescriptions in rural medical institutions is significantly higher (23). Primary medical institutions had limited health resources and a weak diagnostic ability of TB. Physicians were prone to prescribe empirical regimens, resulting in inappropriate use of antibiotics. Studies have confirmed that empirically using FQ antibiotics to treat hypothetical bacterial infections like community-acquired pneumonia or other respiratory diseases, may delay the treatment of TB, and patients may have adverse treatment outcomes due to the emergence of drug-resistant bacteria (24). Therefore, it is very important to strictly control the use of FQs in the treatment of TB in rural areas.

In county-level hospitals such as in Zigui and Yidu, most laboratories had limited ability to culture strains or detect drug resistance. Sputum sample or some cultures were transferred to the municipal-level laboratory for resistance testing. The sample transferring and result feedback usually took a long turn-around time, which made doctors less likely to depend on testing results and to prefer utilizing empirical regimens. Blinded treatment would be the main reason for high burden of drug-resistant TB. A cheap, easy-to-use, rapid molecular testing method of drug resistance is urgently needed in the primary medical institutions for TB diagnosis and treatment. Beyond resistance to the major first-line RIF and INH drugs, FQ resistance also presented a highly concerned problem in Yichang as well.

The genomic-clustering rate of TB clinical strains might reflect the local transmission of TB. We estimated that 30.1% of the bacteria-positive patients were involved in transmission clusters, and the rate was relatively underestimated due the limited sampling proportion. The actual transmission level could be much higher than that in Wuhan (25.5%) (25) and Shenzhen (25.2%) (14), indicating the serosity of TB transmission in rural China. The clustering rates in Yidu and Zigui were 44.6 and 14.7%, respectively. The difference in clustering rates between the two counties might result from varied sampling proportions. Approximately, 30% of bacteria-positive patients from Yidu were enrolled and less than 20% of Zigui patients had whole-genome sequences. The low sampling proportions in Zigui extraordinarily underestimated the actual transmission level and concealed the serious situation of TB transmission. The overall sampling proportions were still not satisfactory, which were considered to be affected by the limited laboratory capacity in the counties, some patients seeking care outside the counties, as well as the lockdowns during the COVID-19 pandemic.

Relatively, by comparing the characteristics of genomic-clustered and non-clustered patient, we identified higher risk of TB transmission among patients less than 45 years old, which was consistent to the findings in other cities that young people had more social contacts and thus increased the transmission risk for infectious diseases (13, 14). Though the cutoff of patients' age differed in these studies and would be smaller in cities, the propensity presented similar for higher transmission risk among younger people. As three quarters of the patients were elder than 50 years, we also calculated the odd ratios using this cut-off, and it was estimated to be 2.31 (1.09–4.88) and 1.89 (0.81–4.40) in univariable and multivariable logistic regression models. The risk was significantly higher for patients aged ≤ 45 compared to those elders, with the adjusted odds ratio of 3.46 (1.28–9.35). Overall, 17.2% (112/654) of all the bacteria-positive patients were in the risk age group, who should be paid higher attention of targeted measures for transmission control.

In conclusion, we revealed the high burden of drug resistance among bacteria-positive TB in Yichang, China, especially the unexpected monoresistance to FQ drugs. Both the high levels of drug-resistant TB and recent transmission alerted the need for enhancement of drug-resistant TB diagnosis and targeted transmission control in primary care of rural areas.

## Data availability statement

The original contributions presented in the study are publicly available. This data can be found here: China National Center for Bio-information [https://ngdc.cncb.ac.cn/~PRJCA013147].

## Ethics statement

The studies involving human participants were reviewed and approved by the Ethics Committee of Yichang Center for Disease Control and Prevention. Written informed consent for participation was not required for this study in accordance with the national legislation and the institutional requirements.

## Author contributions

LJ: conceptualization, methodology, investigation, supervision, and writing—original draft. F-XT: writing—original draft, formal analysis, and visualization. Y-FY: validation, resources, and investigation. J-HL: project administration and supervision. F-HY and C-LB: validation and supervision. Z-YW, X-BY, JM, PiZ, HX, and ZX: data curation. PaZ, ZN, F-QZ, and MC: investigation. QJ and X-JL: writing—review and editing, funding acquisition, and project administration. All authors contributed to the article and approved the submitted version.
