# Supplementary material for: Whole-genome sequencing to characterize the genetic structure and transmission risk of Mycobacterium tuberculosis in Yichang city of China
Source: Front Public Health. 2023 Jan 9;10:1047965. doi: 10.3389/fpubh.2022.1047965 (PMC9868839; doi:10.3389/fpubh.2022.1047965)
Supplement: Supplementary file 1 [file Data_Sheet_1.PDF]

STable 1. Comparison of drug resistance spectrum of different genotypes of tuberculosis

	Total N=161(%)	Beijing type N=69(%)	Non-Beijing type N=92(%)	Significance	Ancient Beijing subtype N=21(%)	Modern Beijing subtype N=71(%)	Significance
**Type of drug resistance**				0.038			0.069
DS	112 (69.6)	57 (82.6)	55 (59.8)	-	11(52.4)	44 (62.0)	-
DR	19 (11.8)	5 (7.2)	14 (15.2)	-	2 (9.5)	12 (16.9)	-
Hr	13 (8.1)	3 (4.3)	10 (10.9)	-	1(4.8)	9 (12.7)	-
Rr	11 (6.8)	2 (2.9)	9 (9.8)	-	5 (23.8)	4 (5.6)	-
MDR	6 (3.7)	2 (2.9)	4 (4.3)	-	2 (9.5)	2 (2.8)	-
**First line anti-tuberculosis drugs**							
INH	19 (11.8)	5 (7.2)	14 (15.2)	n.s.	3 (14.3)	11(15.5)	n.s.
RFP	17 (10.6)	4 (5.8)	13 (14.1)	n.s.	7 (33.3)	6 (8.5)	0.009
EMB	4 (2.5)	0 (0)	4 (4.3)	n.s.	2 (9.5)	2 (2.8)	n.s.
PZA	6 (3.7)	2 (2.9)	4 (4.3)	n.s.	2 (9.5)	2 (2.8)	n.s.
SM	23 (14.3)	7 (10.1)	16 (17.4)	n.s.	2 (9,5)	14 (19.7)	n.s.
**Second line anti-tuberculosis drugs**							
FQs	14 (8.7)	4 (5.8)	10 (10.9)	n.s.	2 (9.5)	8 (11.3)	n.s.
AGs	2 (1.2)	0 (0)	2 (2.2)	n.s.	1 (4.8)	1 (1.4)	n.s.
PAS	6 (3.7)	0 (0)	6 (6.5)	0.038	3 (14.3)	3 (4.2)	n.s.
ETO	7 (4.3)	0 (0)	7 (7.6)	0.020	2 (9.5)	5 (7.0)	n.s.
CS	1 (0.6)	0 (0)	1 (1.1)	n.s.	1 (4.8)	0 (0)	n.s.

Note: n.s., no statistical significance.

STable 2. Demographic and clinical characteristics of treatment-naive and previously treated patients

	Total N=161	Treatment-naive N=139	Previously treated N=22	χ^2	*P* value
**Sex**				0.712	0.399
Female	41 (25.5)	37 (26.6)	4 (18.2)		
Male	120 (74.5)	102 (73.4)	18 (81.8)		
**Age, years**				-	0.194
≤ 45	27 (16.8)	25 (18.0)	2 (9.1)		
46-60	45 (28.0)	37 (26.6)	8 (36.4)		
61-75	64 (39.8)	58 (41.7)	6 (27.3)		
>75	25 (15.5)	19 (13.7)	6 (27.3)		
**Occupation**				-	0.928
Farmer	110 (68.3)	95 (68.3)	15 (68.2)		
Other	11 (6.8)	10 (7.2)	1 (4.5)		
Unemployment	40 (24.8)	34 (24.5)	6 (27.3)		
**Region**				3.468	0.063
Zigui	75 (47.5)	69 (50.4)	6 (28.6)		
Yidu	83 (52.5)	68 (49.6)	15 (71.4)		
**Delayed diagnosis**				-	0.037
≤ 2 weeks	50 (31.8)	38 (28.1)	12 (54.5)		
2-4 weeks	41 (26.1)	39 (28.9)	2 (9.1)		
4-8 weeks	30 (19.1)	28 (20.7)	2 (9.1)		
> 8 weeks	36 (22.9)	30 (22.2)	6 (27.3)		
**Genotype**				0.040	0.843
Beijing	92 (57.1)	79 (56.8)	13 (59.1)		
Euro-American	69 (42.9)	60 (43.2)	9 (40.9)		
**Clustering**				3.611	0.057
Yes	50 (31.1)	47 (33.8)	3 (13.6)		
No	111 (68.9)	92 (66.2)	19 (86.4)		
**RIF resistance**				-	0.015
RIF sensitive	144 (89.4)	128 (92.1)	16 (72.7)		
MDR/RR	17 (10.6)	11 (7.9)	6 (27.3)		
**Sputum smear**				-	0.786
Negative	36 (22.4)	32 (23.0)	4 (18.2)		
Positive	125 (77.6)	107 (77.0)	18 (81.8)		
**Treatment outcome**				9.625	0.002
Favorable	112 (72.7)	102 (77.3)	10 (45.5)		

Other	42 (27.3)	30 (22.7)	12 (54.5)		
**FQs resistance**				-	<0.001
sensitive	147 (91.3)	132(95.0)	15 (68.2)		
resistance	14 (8.7)	7 (5.0)	7 (31.8)		

Note: P value was calculated by the Fisher's exact test if the minimum theoretical frequency in chi-square test was <1.

STable 3. Clustering risk factors of different genotypes and drug-resistant tuberculosis strains

	Genomic-clustered N=50 (%)	Genomic-unique N=111 (%)	χ^2	*P* value
**Genotype**			3.677	0.055
Beijing	23 (46.0)	69 (62.2)		
Euro-American	27 (54.0)	42 (37.8)		
**Type of drug resistance**			1.539	0.854†
DS	34 (68.0)	78 (70.3)		
DR	5 (10.0)	14 (12.6)		
Hr	4 (8.0)	9 (8.1)		
Rr	5 (10.0)	6 (5.4)		
MDR	2 (4.0)	4 (3.6)		
**First line anti-tuberculosis drugs**				
INH	6 (12.0)	13 (11.7)	0.003	0.958
RFP	7 (14.0)	10 (9.0)	0.909	0.340
EMB	0 (0)	4 (3.6)	-	0.311†
PZA	2 (4.0)	4 (3.6)	<0.001	1.000†
**Second line anti-tuberculosis drugs**				
FQs	3 (6.0)	11 (9.9)	-	0.552†
AGs	0 (0)	2 (1.8)	-	1.000†
SM	8 (16.0)	15 (13.5)	0.174	0.677
PAS	4 (8.0)	2 (1.8)	-	0.075†
ETO	0 (0)	7 (6.3)	-	0.100†
CS	0 (0)	1 (0.9)	-	1.000†

† P value was calculated by the Fisher’s exact test if the minimum theoretical frequency in chi-square test was <1.
